# Molecular Diversity of Anthracnose Pathogen Populations Associated with UK Strawberry Production Suggests Multiple Introductions of Three Different *Colletotrichum* Species

**DOI:** 10.1371/journal.pone.0129140

**Published:** 2015-06-18

**Authors:** Riccardo Baroncelli, Antonio Zapparata, Sabrina Sarrocco, Serenella A. Sukno, Charles R. Lane, Michael R. Thon, Giovanni Vannacci, Eric Holub, Surapareddy Sreenivasaprasad

**Affiliations:** 1 School of Life Sciences, Warwick Crop Centre, University of Warwick, Wellesbourne, United Kingdom; 2 Dipartimento di Scienze Agrarie, Alimentari e Agro-ambientali, Università di Pisa, Pisa, Italy; 3 Departamento de Microbiología y Genética, Instituto Hispano-Luso de Investigaciones Agrarias, Universidad de Salamanca, Salamanca, Spain; 4 The Food and Environment Research Agency, York, United Kingdom; 5 Department of Life Sciences, University of Bedfordshire, Luton, United Kingdom; Agriculture and Agri-Food Canada, CANADA

## Abstract

*Fragaria × ananassa* (common name: strawberry) is a globally cultivated hybrid species belonging to *Rosaceae* family. *Colletotrichum acutatum sensu lato *(*s*.*l*.) is considered to be the second most economically important pathogen worldwide affecting strawberries. A collection of 148 *Colletotrichum* spp. isolates including 67 *C*. *acutatum s*.*l*. isolates associated with the phytosanitary history of UK strawberry production were used to characterize multi-locus genetic variation of this pathogen in the UK, relative to additional reference isolates that represent a worldwide sampling of the diversity of the fungus. The evidence indicates that three different species *C*. *nymphaeae*, *C*. *godetiae* and *C*. *fioriniae* are associated with strawberry production in the UK, which correspond to previously designated genetic groups A2, A4 and A3, respectively. Among these species, 12 distinct haplotypes were identified suggesting multiple introductions into the country. A subset of isolates was also used to compare aggressiveness in causing disease on strawberry plants and fruits. Isolates belonging to *C*. *nymphaeae*, *C*. *godetiae* and *C*. *fioriniae* representative of the UK anthracnose pathogen populations showed variation in their aggressiveness. Among the three species, *C*. *nymphaeae* and *C*. *fioriniae* appeared to be more aggressive compared to *C*. *godetiae*. This study highlights the genetic and pathogenic heterogeneity of the *C*. *acutatum s*.*l*. populations introduced into the UK linked to strawberry production.

## Introduction

*Fragaria × ananassa* (common name: strawberry) is a hybrid species cultivated worldwide belonging to the *Rosaceae* family. Since the 1980s, the UK strawberry industry has expanded rapidly representing a significant component of fruit production in the country [1]. Anthracnose is a major disease of cultivated strawberry, caused by two species complexes of the fungus referred to as *C*. *acutatum* and *C*. *gloeosporioides*. *C*. *acutatum* is considered to be the dominant cause of strawberry anthracnose, and the second most important pathogen of strawberry after *Botrytis cinerea* [2–7]. The *C*. *gloeosporioides* complex includes *C*. *fragariae*, which is now considered synonymous with a new species *C*. *theobromicola* [8]. However, researchers have often continued to use the name *C*. *fragariae* when referring to a pathogen that was associated with strawberry anthracnose [9–12]. *C*. *gloeosporioides* is found only occasionally on strawberry in Europe [3,7].

*C*. *acutatum s*.*l*. was described for the first time as a strawberry pathogen in California in 1983 [13], and has since appeared to have spread worldwide, including the UK, through runners and propagating material [2,6,14–16]. A first extensive genetic characterization of *C*. *acutatum s*.*l*. representing the global diversity of the pathogen led to its sub-division into genetic groups named from A1 to A9 [6, 17]. More recently, the *C*. *acutatum s*.*l*. has been sub-divided into more than 30 species based on multi-locus phylogeny [18].

The first record of *C*. *acutatum s*.*l*. in the UK was in 1978, on *Anemone* sp. grown in Jersey [19]. In 1982, the first incidence of anthracnose disease in strawberries caused by *C*. *acutatum s*.*l*. was recorded in the UK, and was attributed to the importation of infected strawberry runners from the USA [20]. DNA sequences in public databases suggest two UK isolates (CBS198.35 and CBS199.35) that were collected in 1935 from the host *Phormium* spp. (common name “New Zealand flax”) belong to *C*. *acutatum s*.*l*. [18, 21]. CABI database records during 1978 to 1983 shows the incidence of the pathogens various hosts and in different locations in the UK (http://www.herbimi.info). However, it seems highly improbable that the first outbreak on strawberry led to the wide dispersal of the pathogen. In 1993, Lovelidge proposed that the continued introduction of infected strawberry material from abroad was so common that the disease was destined to become endemic in the UK [14]. In subsequent years, further outbreaks have been reported on strawberry linked to the importation of infected propagation material mainly from mainland Europe and on other important crop hosts [20,22,23].

Strawberry anthracnose symptoms produced by the two *Colletotrichum* species complexes are similar and can be found on all parts of the plant [12]. Flower blight and fruit rot are common symptoms in the field [24], whereas lesions on stolons, petioles and leaves are mainly found in plant nurseries [15]. Crown symptomatology is characterized by reddish-brown necrotic areas [25] and in some cases stunting and chlorosis have been associated with root necrosis [15].

Research has been carried out to characterize *C*. *acutatum s*.*l*. populations related to strawberry in specific geographic areas including Israel, France, Bulgaria, Spain, Belgium and other European countries [2–5,7,26] and from specific regions of the USA [25]. Other research has attempted to characterize *C*. *acutatum s*.*l*. related to strawberry using isolates collected worldwide [3], both by genomic fingerprinting (such as RFLP, apPCR, etc.) and sequence analysis based on the ITS region. Results have highlighted the presence of at least one representative “clonal” population suggesting a single source of origin and, consequentially, that the disease is spread through infected propagation material. However, ITS sequences alone or genomic fingerprinting are not suitable to discriminate among the newly assigned species designations.

In a recent study based on the analysis of more than two decades of anthracnose incidence data sets gathered by authorities responsible for plant health, trade was identified as the main route of entry and establishment of *C*. *acutatum* in the UK strawberry production. Over this period, various nurseries were importing planting material into the UK, and at least 55 cases of infested material that was planted in the field through imports that were not intercepted by the border inspection posts, were identified [20].

The focus of the present study was to assess the extent of the genetic and pathogenic diversity of these introduced pathogen populations mainly utilising a unique collection of *C*. *acutatum s*. *l*. isolates established through the plant health inspection surveys from the early 1980s onwards. We focused on *C*. *acutatum s*.*l*. because previous reports from France, Israel, UK, Bulgaria and Spain had described this taxa as a major widely distributed pathogen, compared with other species such as *C*. *gloeosporioides s*.*l*. that occur less frequently in Europe [2–5,12]. A range of historic and contemporary *C*. *acutatum s*. *l*. isolates including those from worldwide strawberry crops, other plant hosts in the UK, as well as worldwide representatives from different hosts building on our previous work were accessed as reference sources for determining the genetic and species identities of isolates associated with UK strawberry anthracnose phytosanitary control work. Based on multi-locus phylogenetic analysis, we have identified 12 different haplotypes that belong to three different species *C*. *nymphaeae*, *C*. *godetiae* and *C*. *fioriniae* suggesting multiple introductions of the strawberry anthracnose pathogen. Pathogenic and growth characteristics of these haplotype representatives further highlight the heterogeneity of the introduced pathogen populations.

## Materials and Methods

### Fungal isolates and culture conditions

**Fig 1:**
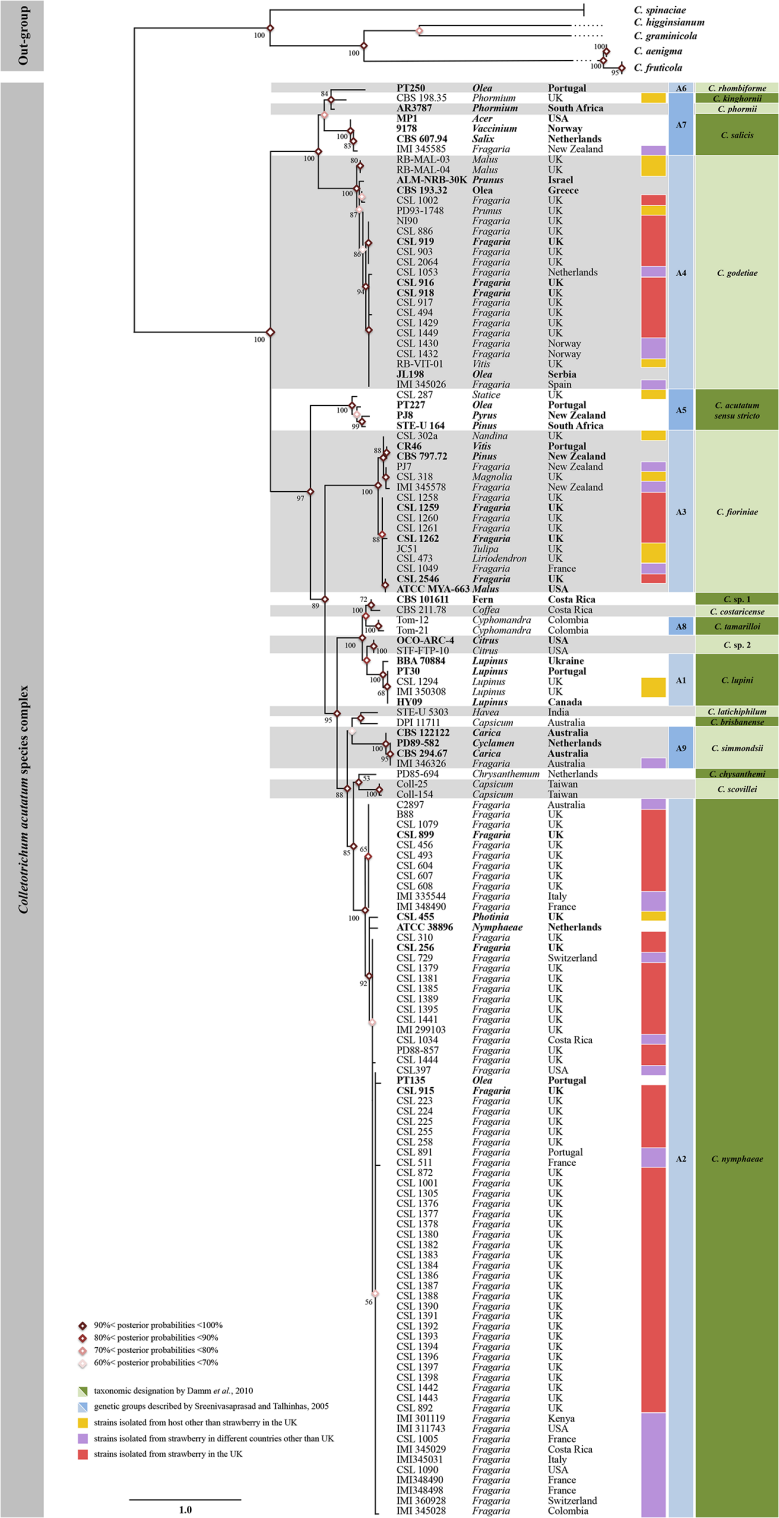
Multilocus phylogenetic analysis of the *Colletotrichum* isolates used in this study. Bayesian MCMC analysis tree constructed from the alignment based on the concatenation of rRNA, TUB, MAT1-2 and GPDH partial sequences of 140 *Colletotrichum acutatum sensu lato* isolates used in this study. The tree was rooted with sequences from *C*. *graminicola* and *C*. *higginsianum* retrieved from whole genome sequences and sequences of four *C*. *gloeosporioides sensu lato* and two *C*. *spinaciae* obtained experimentally. Isolates used to investigate variation in aggressiveness are highlighted in bold.

A diverse collection of *C*. *acutatum s*.*l*. was assembled for this study including: 67 isolates associated with strawberry production in the UK (obtained from the UK Food and Environment Research Agency, or FERA responsible for plant health within the Department for Environment, Food and Rural Affairs), 27 *C*. *acutatum s*.*l*. isolates collected from strawberry in other countries, and 13 isolates collected from other host species in the UK. For further comparison, 33 isolates were added to represent other genetic groups, and novel species from previous studies [6,17,18]. This included two isolates of *C*. *fruticola*, two isolates of *C*. *aenigma* (belonging to *C*. *gloeosporioides* species complex [8]) associated with strawberry, two UK isolates of *C*. *spinaciae* and one isolate each of *C*. *graminicola*, *C*. *higginsianum* [27] and *C*. *fioriniae* [28]. Sequence data of the markers was retrieved from the reference genome sequences available from Genbank for *C*. *graminicola* and *C*. *higginsianum* (accession numbers: ACOD01000000 and CACQ02000000, respectively) used among out-groups in the phylogenetic analysis (Fig 1). Details of the isolate collection used in the present study are provided in Table 1.

**Table 1:** *Colletotrichum* sp. strains used in this study with isolation details and GenBank accessions.

Strain Code	Genus	Species	Genetic group [6]	Country	Host	Accession numbers			
** **	** **	** **	** **	** **	** **	ITS	TUB	MAT1-2	GAPDH
***Isolates from strawberry in UK***		*** ***	*** ***	*** ***	*** ***	*** ***	*** ***	*** ***	*** ***
**B88**	*Colletotrichum*	*nymphaeae*	A2	United Kingdom	*Fragaria x ananassa*	KM246514	KM251867	KM251969	KM252115
**NI90**	*Colletotrichum*	*godetiae*	A4	United Kingdom	*Fragaria x ananassa*	AF411766	AJ409294	KM251970	KM252116
**CSL 1079**	*Colletotrichum*	*nymphaeae*	A2	United Kingdom	*Fragaria x ananassa*	KM246515	KM251868	KM251981	KM252118
**CSL 2546**	*Colletotrichum*	*fioriniae*	A3	United Kingdom	*Fragaria x ananassa*	KM246516	KM251870	KM251983	KM252120*
**CSL 899**	*Colletotrichum*	*nymphaeae*	A2	United Kingdom	*Fragaria x ananassa*	KM246518	KM251872	KM251985	KM252122*
**CSL 310**	*Colletotrichum*	*nymphaeae*	A2	United Kingdom	*Fragaria x ananassa*	KM246519	KM251873	KM251986	KM252123
**CSL 915**	*Colletotrichum*	*nymphaeae*	A2	United Kingdom	*Fragaria x ananassa*	KM246520	KM251874	KM251987	KM252124*
**CSL 886**	*Colletotrichum*	*godetiae*	A4	United Kingdom	*Fragaria x ananassa*	KM246521	KM251875	KM251988	KM252125
**CSL 919**	*Colletotrichum*	*godetiae*	A4	United Kingdom	*Fragaria x ananassa*	KM246522	KM251876	KM251989	KM252126*
**CSL 916**	*Colletotrichum*	*godetiae*	A4	United Kingdom	*Fragaria x ananassa*	KM246523	KM251877	KM251990	KM252127*
**CSL 918**	*Colletotrichum*	*godetiae*	A4	United Kingdom	*Fragaria x ananassa*	KM246524	KM251878	KM251991	KM252128*
**CSL 917**	*Colletotrichum*	*godetiae*	A4	United Kingdom	*Fragaria x ananassa*	KM246525	KM251879	KM251992	KM252129
**CSL 223**	*Colletotrichum*	*nymphaeae*	A2	United Kingdom	*Fragaria x ananassa*	KM246526	KM251880	KM251993	KM252130
**CSL 224**	*Colletotrichum*	*nymphaeae*	A2	United Kingdom	*Fragaria x ananassa*	KM246527	KM251881	KM251994	KM252131
**CSL 225**	*Colletotrichum*	*nymphaeae*	A2	United Kingdom	*Fragaria x ananassa*	KM246528	KM251882	KM251995	KM252132
**CSL 255**	*Colletotrichum*	*nymphaeae*	A2	United Kingdom	*Fragaria x ananassa*	KM246529	KM251883	KM251996	KM252133
**CSL 256**	*Colletotrichum*	*nymphaeae*	A2	United Kingdom	*Fragaria x ananassa*	KM246530	KM251884	KM251997	KM252134*
**CSL 258**	*Colletotrichum*	*nymphaeae*	A2	United Kingdom	*Fragaria x ananassa*	KM246531	KM251885	KM251998	KM252135
**CSL 456**	*Colletotrichum*	*nymphaeae*	A2	United Kingdom	*Fragaria vesca*	KM246532	KM251886	KM251999	KM252136
**CSL 493**	*Colletotrichum*	*nymphaeae*	A2	United Kingdom	*Fragaria x ananassa*	KM246533	KM251887	KM252000	KM252137
**CSL 494**	*Colletotrichum*	*godetiae*	A4	United Kingdom	*Fragaria vesca*	KM246534	KM251888	KM252001	KM252138
**CSL 604**	*Colletotrichum*	*nymphaeae*	A2	United Kingdom	*Fragaria x ananassa*	KM246535	KM251890	KM252003	KM252140
**CSL 607**	*Colletotrichum*	*nymphaeae*	A2	United Kingdom	*Fragaria x ananassa*	KM246538	KM251893	KM252006	KM252143
**CSL 608**	*Colletotrichum*	*nymphaeae*	A2	United Kingdom	*Fragaria x ananassa*	KM246539	KM251894	KM252007	KM252144
**CSL 872**	*Colletotrichum*	*nymphaeae*	A2	United Kingdom	*Fragaria x ananassa*	KM246541	KM251896	KM252009	KM252146
**CSL 903**	*Colletotrichum*	*godetiae*	A4	United Kingdom	*Fragaria x ananassa*	KM246542	KM251897	KM252010	KM252147
**CSL 1001**	*Colletotrichum*	*nymphaeae*	A2	United Kingdom	*Fragaria x ananassa*	KM246543	KM251898	KM252011	KM252148
**CSL 1258**	*Colletotrichum*	*fioriniae*	A3	United Kingdom	*Fragaria x ananassa*	KM246544	KM251899	KM252012	KM252149
**CSL 1259**	*Colletotrichum*	*fioriniae*	A3	United Kingdom	*Fragaria x ananassa*	KM246545	KM251900	KM252013	KM252150*
**CSL 1260**	*Colletotrichum*	*fioriniae*	A3	United Kingdom	*Fragaria x ananassa*	KM246546	KM251901	KM252014	KM252151
**CSL 1261**	*Colletotrichum*	*fioriniae*	A3	United Kingdom	*Fragaria x ananassa*	KM246547	KM251902	KM252015	KM252152
**CSL 1262**	*Colletotrichum*	*fioriniae*	A3	United Kingdom	*Fragaria x ananassa*	KM246548	KM251903	KM252016	KM252153*
**CSL 1305**	*Colletotrichum*	*nymphaeae*	A2	United Kingdom	*Fragaria x ananassa*	KM246549	KM251904	KM252017	KM252154
**CSL 1376**	*Colletotrichum*	*nymphaeae*	A2	United Kingdom	*Fragaria x ananassa*	KM246550	KM251905	KM252018	KM252155
**CSL 1377**	*Colletotrichum*	*nymphaeae*	A2	United Kingdom	*Fragaria x ananassa*	KM246551	KM251906	KM252019	KM252156
**CSL 1378**	*Colletotrichum*	*nymphaeae*	A2	United Kingdom	*Fragaria x ananassa*	KM246552	KM251907	KM252020	KM252157
**CSL 1379**	*Colletotrichum*	*nymphaeae*	A2	United Kingdom	*Fragaria x ananassa*	KM246553	KM251908	KM252021	KM252158
**CSL 1380**	*Colletotrichum*	*nymphaeae*	A2	United Kingdom	*Fragaria x ananassa*	KM246554	KM251909	KM252022	KM252159
**CSL 1381**	*Colletotrichum*	*nymphaeae*	A2	United Kingdom	*Fragaria x ananassa*	KM246555	KM251910	KM252023	KM252160
**CSL 1382**	*Colletotrichum*	*nymphaeae*	A2	United Kingdom	*Fragaria x ananassa*	KM246556	KM251911	KM252024	KM252161
**CSL 1383**	*Colletotrichum*	*nymphaeae*	A2	United Kingdom	*Fragaria x ananassa*	KM246557	KM251912	KM252025	KM252162
**CSL 1384**	*Colletotrichum*	*nymphaeae*	A2	United Kingdom	*Fragaria x ananassa*	KM246558	KM251913	KM252026	KM252163
**CSL 1385**	*Colletotrichum*	*nymphaeae*	A2	United Kingdom	*Fragaria x ananassa*	KM246559	KM251914	KM252027	KM252164
**CSL 1386**	*Colletotrichum*	*nymphaeae*	A2	United Kingdom	*Fragaria x ananassa*	KM246560	KM251915	KM252028	KM252165
**CSL 1387**	*Colletotrichum*	*nymphaeae*	A2	United Kingdom	*Fragaria x ananassa*	KM246561	KM251916	KM252029	KM252166
**CSL 1388**	*Colletotrichum*	*nymphaeae*	A2	United Kingdom	*Fragaria x ananassa*	KM246562	KM251917	KM252030	KM252167
**CSL 1389**	*Colletotrichum*	*nymphaeae*	A2	United Kingdom	*Fragaria x ananassa*	KM246563	KM251918	KM252031	KM252168
**CSL 1390**	*Colletotrichum*	*nymphaeae*	A2	United Kingdom	*Fragaria x ananassa*	KM246564	KM251919	KM252032	KM252169
**CSL 1391**	*Colletotrichum*	*nymphaeae*	A2	United Kingdom	*Fragaria x ananassa*	KM246565	KM251920	KM252033	KM252170
**CSL 1392**	*Colletotrichum*	*nymphaeae*	A2	United Kingdom	*Fragaria x ananassa*	KM246566	KM251921	KM252034	KM252171
**CSL 1393**	*Colletotrichum*	*nymphaeae*	A2	United Kingdom	*Fragaria x ananassa*	KM246567	KM251922	KM252035	KM252172
**CSL 1394**	*Colletotrichum*	*nymphaeae*	A2	United Kingdom	*Fragaria x ananassa*	KM246568	KM251923	KM252036	KM252173
**CSL 1395**	*Colletotrichum*	*nymphaeae*	A2	United Kingdom	*Fragaria x ananassa*	KM246569	KM251924	KM252037	KM252174
**CSL 1396**	*Colletotrichum*	*nymphaeae*	A2	United Kingdom	*Fragaria x ananassa*	KM246570	KM251925	KM252038	KM252175
**CSL 1397**	*Colletotrichum*	*nymphaeae*	A2	United Kingdom	*Fragaria x ananassa*	KM246571	KM251926	KM252039	KM252176
**CSL 1398**	*Colletotrichum*	*nymphaeae*	A2	United Kingdom	*Fragaria x ananassa*	KM246572	KM251927	KM252040	KM252177
**CSL 1429**	*Colletotrichum*	*godetiae*	A4	United Kingdom	*Fragaria x ananassa*	KM246573	KM251928	KM252041	KM252178
**CSL 1441**	*Colletotrichum*	*nymphaeae*	A2	United Kingdom	*Fragaria x ananassa*	KM246574	KM251929	KM252042	KM252179
**CSL 1442**	*Colletotrichum*	*nymphaeae*	A2	United Kingdom	*Fragaria x ananassa*	KM246575	KM251930	KM252043	KM252180
**CSL 1443**	*Colletotrichum*	*nymphaeae*	A2	United Kingdom	*Fragaria x ananassa*	KM246576	KM251931	KM252044	KM252181
**CSL 1444**	*Colletotrichum*	*nymphaeae*	A2	United Kingdom	*Fragaria x ananassa*	KM246577	KM251932	KM252045	KM252182
**CSL 1449**	*Colletotrichum*	*godetiae*	A4	United Kingdom	*Fragaria x ananassa*	KM246578	KM251933	KM252046	KM252183
**CSL 2064**	*Colletotrichum*	*godetiae*	A4	United Kingdom	*Fragaria x ananassa*	KM246579	KM251934	KM252047	KM252184
**CSL 1002**	*Colletotrichum*	*godetiae*	A4	United Kingdom	*Fragaria x ananassa*	KM246580	KM251935	KM252048	KM252185
**CSL 892**	*Colletotrichum*	*nymphaeae*	A2	United Kingdom	*Fragaria x ananassa*	KM246584	KM251938	KM252053	KM252188
**IMI 299103** [18]	*Colletotrichum*	*nymphaeae*	A2	United Kingdom	*Fragaria vesca*	JQ948231	JQ949882	KM252069	JQ948561
**PD88-857**, CBS 125973 [18]	*Colletotrichum*	*nymphaeae*	A2	United Kingdom	*Fragaria x ananassa*	JQ948232	JQ949883	KM252100	JQ948562
**C. acutatum sensu lato *from strawberry worldwide***			** **	** **	** **	** **	** **	** **	** **
**C2897**	*Colletotrichum*	*nymphaeae*	A2	Australia	*Fragaria x ananassa*	AJ300558	AJ314718	KM251967	KM252113
**CSL 397**	*Colletotrichum*	*nymphaeae*	A2	USA	*Fragaria x ananassa*	AF411765	AJ409296	KM251968	KM252114
**CSL 1053**	*Colletotrichum*	*godetiae*	A4	Netherlands	*Fragaria x ananassa*	AJ536210	KM251869	KM251982	KM252119
**CSL 891**	*Colletotrichum*	*nymphaeae*	A2	Portugal	*Fragaria sp*.	EF622184	KM251889	KM252002	KM252139
**CSL 511**	*Colletotrichum*	*nymphaeae*	A2	France	*Fragaria x ananassa*	KM246536	KM251891	KM252004	KM252141
**CSL 729**	*Colletotrichum*	*nymphaeae*	A2	Switzerland	*Fragaria x ananassa*	KM246537	KM251892	KM252005	KM252142
**CSL 1430**	*Colletotrichum*	*godetiae*	A4	Norway	*Fragaria vesca*	KM246585	KM251939	KM252054	KM252189
**CSL 1432**	*Colletotrichum*	*godetiae*	A4	Norway	*Fragaria x ananassa*	KM246586	KM251940	KM252055	KM252190
**PJ7** [28]	*Colletotrichum*	*fioriniae*	A3	New Zealand	*Fragaria x ananassa*	genome: JARH00000000			
**CSL 1020**, IMI 301119 [18]	*Colletotrichum*	*nymphaeae*	A2	Kenya	*Fragaria vesca*	JQ948266	JQ949917	KM252070	JQ948596
**IMI 311743** [18]	*Colletotrichum*	*nymphaeae*	A2	USA	*Fragaria x ananassa*	JQ948258	JQ949909	KM252071	JQ948588
**IMI 335544**	*Colletotrichum*	*nymphaeae*	A2	Italy	*Fragaria x ananassa*	KJ018636	KJ018648	KM252072	KJ018660
**IMI 345026** **[18]**	*Colletotrichum*	*godetiae*	A4	Spain	*Fragaria x ananassa*	JQ948424	JQ950075	KM252073	JQ948755
**CSL 1005**, IMI 345027	*Colletotrichum*	*nymphaeae*	A2	France	*Fragaria x ananassa*	AJ536199	KM251946	KM252074	KM252198
**IMI 345028**	*Colletotrichum*	*nymphaeae*	A2	Colombia	*Fragaria x ananassa*	AF090853	KM251947	KM252075	KM252199
**IMI 345029**	*Colletotrichum*	*nymphaeae*	A2	Costa Rica	*Fragaria x ananassa*	KM246591	KM251948	KM252076	KM252200
**CSL 1034,** IMI345030	*Colletotrichum*	*nymphaeae*	A2	Costa Rica	*Fragaria x ananassa*	AJ536203	KM251949	KM252077	KM252201
**IMI 345031**	*Colletotrichum*	*nymphaeae*	A2	Italy	*Fragaria x ananassa*	KM246592	KM251950	KM252078	KM252202
**IMI 345578** [18]	*Colletotrichum*	*fioriniae*	A3	New Zealand	*Fragaria ananassa*	JQ948334	JQ949985	KM252080	JQ948664
**CSL 1046**, IMI 346326	*Colletotrichum*	*simmondsii*	A2	Australia	*Fragaria x ananassa*	AJ536208	KM251952	KM252081	KM252204
**IMI 345585** [18]	*Colletotrichum*	*salicis*	A7	New Zealand	*Fragaria x ananassa*	JQ948476	JQ950127	KM252084	JQ948807
**CSL 1090**, IMI 348160	*Colletotrichum*	*nymphaeae*	A2	USA	*Fragaria x ananassa*	AJ536200	KM251953	KM252086	KM252205
**IMI 348177** [18]	*Colletotrichum*	*nymphaeae*	A2	USA	*Fragaria x ananassa*	KM246593	KM251954	KM252087	KM252206
**IMI 348490**	*Colletotrichum*	*nymphaeae*	A2	France	*Fragaria x ananassa*	KM246594	KM251955	KM252088	KM252207
**CSL 1086**, IMI 348498	*Colletotrichum*	*nymphaeae*	A2	France	*Fragaria x ananassa*	KM246595	KM251956	KM252089	KM252208
**CSL 1049**, IMI 348499	*Colletotrichum*	*fioriniae*	A3	France	*Fragaria x ananassa*	AJ536220	KM251957	KM252090	KM252209
**IMI 360928** [18]	*Colletotrichum*	*nymphaeae*	A2	Switzerland	*Fragaria x ananassa*	JQ948243	JQ949894	KM252091	JQ948573
***Strains isolated from different hosts in UK***		*** ***	*** ***	*** ***	*** ***	*** ***	*** ***	*** ***	*** ***
**RB-MAL-03** [23]	*Colletotrichum*	*godetiae*	A4	United Kingdom	*Malus domestica*	KF834206	KF834207	KM252049	KF834208
**RB-MAL-04**	*Colletotrichum*	*godetiae*	A4	United Kingdom	*Malus domestica*	KM246582	KM251936	KM252050	KM252186
**CSL 1294**	*Colletotrichum*	*lupini*	A1	United Kingdom	*Lupinus polyphyllus*	AJ300561	KM251944	KM252059	KM252194
**CSL 287** [18]	*Colletotrichum*	*acutatum*	A5	United Kingdom	*Statice sp*.	JQ948389	JQ950040	KM252060	JQ948720
**RB-VIT-01**,CBS 129951 [22]	*Colletotrichum*	*godetiae*	A4	United Kingdom	*Vitis vinifera*	KF834203	KF834204	KM252061	KF834205
**CSL 455** [18]	*Colletotrichum*	*nymphaeae*	A2	United Kingdom	*Photinia sp*.	JQ948217	JQ949868	KM252063	JQ948547
**JC51**, CBS 129948 [18]	*Colletotrichum*	*fioriniae*	A3	United Kingdom	*Tulipa sp*.	AJ749680	KM251945	KM252064	KM252195
**CSL 302a**	*Colletotrichum*	*fioriniae*	A3	United Kingdom	*Nandina domestica*	AJ749670	AJ748626	KM252065	KM252196
**CSL 473** [18]	*Colletotrichum*	*fioriniae*	A3	United Kingdom	*Liriodendron tulipifera*	JQ948345	JQ949996	KM252066	JQ948675
**CSL 318** [18]	*Colletotrichum*	*fioriniae*	A3	United Kingdom	*Magnolia sp*.	JQ948346	JQ949997	KM252067	JQ948676
**IMI 350308**	*Colletotrichum*	*lupini*	A1	United Kingdom	*Lupinus sp*.	AJ300561	KM251951	KM252079	KM252203
**CBS 198.35** [18]	*Colletotrichum*	*kinghornii*	A7	United Kingdom	*Phormium sp*.	JQ948454	JQ950105	KM252083	JQ948785
**PD93-1748**, CBS 126527 [18]	*Colletotrichum*	*godetiae*	A4	United Kingdom	*Prunus avium*	JQ948408	JQ950059	KM252101	JQ948739
***Isolates from different host worldwide and used as references for genetics groups / species***					*** ***	*** ***	*** ***	*** ***	*** ***
**PT250**, CBS 129953 [18]	*Colletotrichum*	*rhombiforme*	A6	Portugal	*Olea europaea*	JQ948457	JQ950108	KM251971	JQ948788*
**PT135**, CBS 129945 [18]	*Colletotrichum*	*nymphaeae*	A2	Portugal	*Olea europaea*	JQ948201	JQ949852	KM251972	JQ948531
**PD85-694**, CBS 126519 [18]	*Colletotrichum*	*chrysanthemi*	A2	Netherlands	*Chrysanthemum* sp.	JQ948272	JQ949923	KM251973	JQ948602
**PD89-582**, CBS 126524 [18]	*Colletotrichum*	*simmondsii*	A2	Netherlands	*Cyclamen* sp.	JQ948281	JQ949932	KM251974	JQ948611*
**PT227**, CBS 129952 [18]	*Colletotrichum*	*acutatum*	A5	Portugal	*Olea europaea*	JQ948364	JQ950015	KM251975	JQ948695*
**Tom-21**, CBS 129954 [18]	*Colletotrichum*	*tamarilloi*	A8	Colombia	*Cyphomandra betacea*	JQ948188	JQ949839	KM251976	JQ948518
**Tom-12**, CBS 129955 [18]	*Colletotrichum*	*tamarilloi*	A8	Colombia	*Cyphomandra betacea*	JQ948189	JQ949840	KM251977	JQ948519
**CBS 193.32** [18]	*Colletotrichum*	*godetiae*	A4	Greece	*Olea europaea*	JQ948415	JQ950066	KM251978	JQ948746*
**PT30**	*Colletotrichum*	*lupini*	A1	Portugal	*Lupinus albus*	AJ300561	AJ292250	KM251979	KM252117*
**CR46**, CBS 129947 [18]	*Colletotrichum*	*fioriniae*	A3	Portugal	*Vitis vinifera*	JQ948343	JQ949994	KM251980	JQ948673*
**9178**	*Colletotrichum*	*salicis*	A7	Norway	*Vaccinium corymbosum*	KM246583	KM251937	KM252051	KM252187*
**MP1**, CBS 129972 [18]	*Colletotrichum*	*salicis*	A7	USA	*Acer platanoides*	JQ948466	JQ950117	KM252052	JQ948797*
**PJ8**	*Colletotrichum*	*acutatum*	A5	New Zealand	*Pyrus pyrifolia*	KM246587	KM251941	KM252056	KM252191*
**ATCC MYA-663**	*Colletotrichum*	*fioriniae*	A3	USA	*Malus domestica*	KM246589	KM251943	KM252058	KM252193*
**HY09**	*Colletotrichum*	*lupini*	A1	Canada	*Lupinus albus*	KJ018635	KJ018647	KM252062	KJ018659*
**JL198**	*Colletotrichum*	*godetiae*	A4	Serbia	*Olea europaea*	AJ749689	AJ748613	KM252068	KM252197*
**AR3787**, CBS 118191 [18]	*Colletotrichum*	*phormii*	A7	South Africa	*Phormium sp*.	JQ948453	JQ950104	KM252082	JQ948784*
**CBS 607.94** [18]	*Colletotrichum*	*salicis*	A7	Netherlands	*Salix sp*.	JQ948460	JQ950111	KM252085	JQ948791*
**ALM-NRB-30K**	*Colletotrichum*	*godetiae*	A4	Israel	*Prunus dulcis*	DQ003129	KM251960	KM252094	KM252212*
**CBS 101611** [18]	*Colletotrichum*	sp. 1	-	Costa Rica	Fern	JQ948196	JQ949847	KM252095	JQ948526*
**BBA 70884**, CBS 109225 [18]	*Colletotrichum*	*lupini*	A1	Ukraine	*Lupinus albus*	JQ948155	JQ949806	KM252096	JQ948485*
**STE-U 164**, CBS 112980 [18]	*Colletotrichum*	*acutatum*	A5	South Africa	*Pinus radiata*	JQ948356	JQ950007	KM252097	JQ948687*
**STE-U 5303**, CBS 112989 [18]	*Colletotrichum*	*laticiphilum*	A2	India	*Hevea brasiliensis*	JQ948289	JQ949940	KM252098	JQ948619
**CBS 122122** [18]	*Colletotrichum*	*simmondsii*	A2	Australia	*Carica papaya*	JQ948276	JQ949927	KM252099	JQ948606*
**CBS 211.78** [18]	*Colletotrichum*	*costaricense*	-	Costa Rica	*Coffea sp*.	JQ948181	JQ949832	KM252102	JQ948511
**DPI 11711**, CBS 292.67 [18]	*Colletotrichum*	*brisbanense*	A2	Australia	*Capsicum annuum*	JQ948291	JQ949942	KM252103	JQ948621
**DPI 13483**, CBS 294.67 [18]	*Colletotrichum*	*simmondsii*	A2	Australia	*Carica papaya*	JQ948277	JQ949928	KM252104	JQ948607*
**ATCC 38896**, CBS 526.77 [18]	*Colletotrichum*	*nymphaeae*	A2	Netherlands	*Nymphaeae alba*	JQ948199	JQ949850	KM252105	JQ948529
**CBS 797.72**	*Colletotrichum*	*fioriniae*	A3	New Zealand	*Pinus radiata*	KM246598	KM251961	KM252106	KM252213*
**OCO-ARC-4**	*Colletotrichum*	sp. 2	-	USA	*Citrus x sinensis*	EU647305	KM251962	KM252107	EU647318*
**STF-FTP-10**	*Colletotrichum*	sp. 2	-	USA	*Citrus x sinensis*	EU647306	KM251963	KM252108	EU647319
**Coll-25**	*Colletotrichum*	*scovillei*	A2	Taiwan	*Capsicum annum*	KJ018637	KJ018649	KM252109	KJ018661
**Coll-154**	*Colletotrichum*	*scovillei*	A2	Taiwan	*Capsicum annum*	DQ410028	KM251964	KM252110	KM252214
***Isolates as out-group***	*** ***	*** ***	*** ***	*** ***	*** ***	*** ***	*** ***	*** ***	*** ***	*** ***
CSL 311	*Colletotrichum*	*fruticola*	OG	USA	*Fragaria x ananassa*	KM246512	KM251865	KM251965	KM252111*
CSL 386	*Colletotrichum*	*fruticola*	OG	USA	*Fragaria x ananassa*	KM246513	KM251866	KM251966	KM252112*
CSL 780	*Colletotrichum*	*aenigma*	OG	UK	*Fragaria x ananassa*	KM246517	KM251871	KM251984	KM252121*
CSL 869	*Colletotrichum*	*aenigma*	OG	UK	*Fragaria x ananassa*	KM246540	KM251895	KM252008	KM252145*
CSL 593	*Colletotrichum*	*spinaciae*	OG	UK	*Spinacia oleracea*	KM246596	KM251958	KM252092	KM252210
CSL 739	*Colletotrichum*	*spinaciae*	OG	UK	*Spinacia oleracea*	KM246597	KM251959	KM252093	KM252211
M1.001 [27]	*Colletotrichum*	*graminicola*	OG	USA	*Zea mais*	genome: ACOD0100000000			
IMI 349063 [27]	*Colletotrichum*	*higginsianum*	OG	Trinidad and Tobago	*Brassica chinensis*	genome: CACQ0200000000			

Abbreviation

CBS: Culture collection of the Centraalbureau voor Schimmelcultures, Fungal Biodiversity Centre, Utrecht, The Netherlands IMI: Culture collection of CABI Europe UK Centre, Egham, UK CSL: Culture collection of The Food and Eviroment Research Agency, DEFRA, York, UK OG: out-group* strains used for pathogenicity tests

Cultures were maintained at 25°C on potato dextrose agar medium (PDA, Difco Laboratories, USA) for up to ten days under a 12 h light/ 12 h dark cycle. Long–term storage at 4°C involved cutting mycelial plugs from the edge of actively growing cultures on PDA and suspending them in sterile water.

### Characterization of genetic variation

Genomic DNA was extracted according to the Chelex 100 protocol [29], with some modifications [30]. DNA was quantified using a NanoDrop ND-1000 spectrophotometer (Thermo Scientific, DE, USA).

Various target regions were used to characterise genetic diversity amongst the fungal isolates including: ITS region, partial sequence of the beta-tubulin 2 gene (TUB) (exons 3 through 6, including introns 2 through 4), partial sequence of the glyceraldehyde-3-phosphate dehydrogenase (GAPDH) gene, and partial sequence of the mating type gene (MAT1-2) (the intron included in the conserved HMGbox region). Target regions were amplified using PCR reaction mixes (20 μl) that contained 1 μl of DNA, 1 μl each of primer (20 μM), 7 μl of H_2_0 and 10 μl of ReadyMix RedTaq (Sigma).

PCR amplification of the target regions for sequencing was carried out as described below using previously published primers under conditions standardised for routine work. For ITS, primers ITS1Ext and ITS4Ext [31] were used. The amplification program consisted of 2 min of initial denaturation (95°C), 30 cycles of amplification (1 min at 94°C, 1 min at 55°C, and 1 min at 72°C) and a final extension at 72°C for 5 min. For TUB, primers TB5 and TB6 [31] were used. The amplification program consisted of 2 min initial denaturation (95°C), 30 cycles of amplification (1 min at 94°C, 1 min at 65°C and 1 min at 72°C) and a final extension at 72°C for 5 min. For GAPDH, primers GDF1 and GDR1 [32] were used. The amplification program consisted of 2 min initial denaturation at 95°C, 35 cycles of amplification (1 min at 94°C, 1 min at 60°C and 30 sec at 72°C) and a final extension at 72°C for 3 min. For MAT1-2, primers HMGacuF2 and HMGacuR [21] for *C*. *acutatum s*.*l*. and primers HMGgloeF1 and HMGgloeR1 for *C*. *gloeosporioides s*. *l*. [33] were used. The amplification program consisted of 5 min initial denaturation at 95°C, 40 cycles of amplification (1 min at 95°C, 1 min between 48°C and 55°C and 30s at 72°C) and a final extension of 20 min at 72°C. PCR products were separated using gel electrophoresis and purified using the QIAquick PCR purification kit (Qiagen, USA).

Sequencing of PCR products was carried out at the University of Warwick Genomics Centre, using an ABI Prism 7900HT or ABI3100 sequence detection system (Applied Biosystems, UK). PCR products were cleaned up and then quantified with reference to a ladder (Bioline EasyLadder I) containing DNA fragments of known concentration. One to five microliters of each sample (depending on DNA concentration) were used in sequencing reactions with the BigDye Terminator v3.1 cycle sequencing kit (Applied Biosystems, UK). ABI trace files were analyzed and consensus sequences were generated using Geneious 7.1.6 [34]. All the sequences were aligned using MUSCLE (http://www.ebi.ac.uk/Tools/msa/muscle/) and were manually edited to optimise the alignment, as required. Multiple alignments were end trimmed in order to have comparable nucleotides.

**Fig 2:**
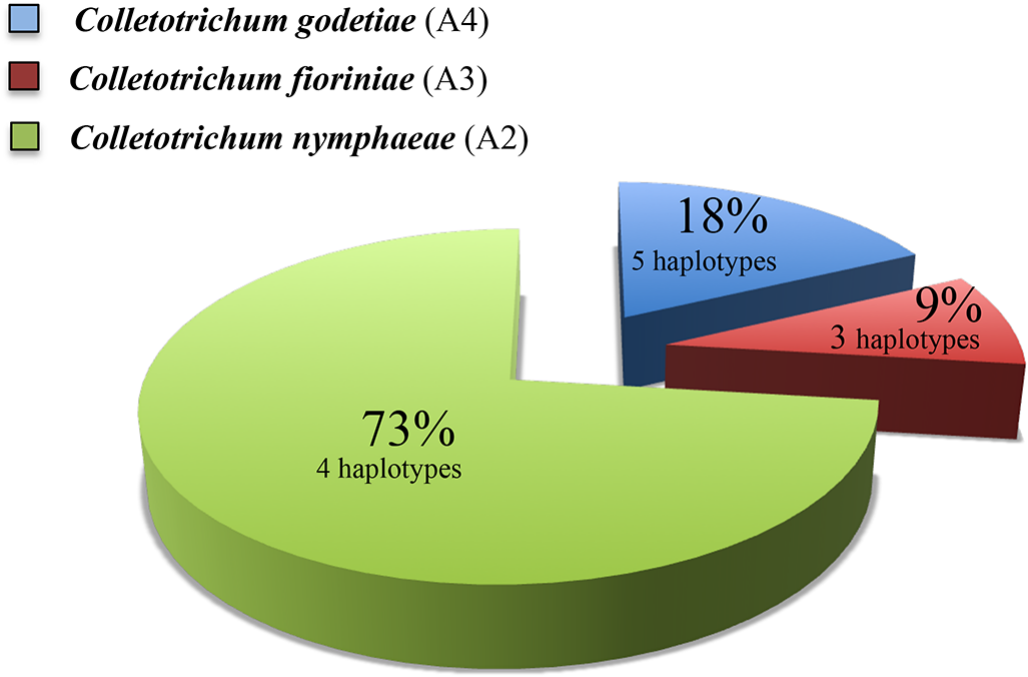
Percentage occurrence of *Colletotrichum acutatum sensu lato* species and relative numbers of haplotypes identified among 67 strains isolated from strawberry in UK.

Multiple sequence alignments were exported to MEGA5 [35] where best-fit substitution models were calculated for each separate sequence dataset. In order to evaluate whether the four sequenced loci were congruent and suitable for concatenation, tree topologies of 50% Neighbour-Joining bootstrap and maximum parsimony analysis (100,000 replicates) were separately performed for each gene and visually compared [36]. The multilocus concatenated alignment (ITS, TUB2, MAT1-2 and GAPDH) was performed with Geneious 7.1.6 [34]. A Markov Chain Monte Carlo (MCMC) algorithm was used to generate phylogenetic trees with Bayesian probabilities using MrBayes 3.2.1 [37] for combined sequence datasets. Models of nucleotide substitution for each gene determined by MEGA5 were included for each locus. The analysis in MrBayes ran for 5000000 of generations to reach a P value lower than 0.01 with two parallel searches using three heated and one cold Markov chain sampled every 100 generations; 25% of generations were discarded as burn-in. Further phylogenetic analysis was performed by the neighbour-joining method with 1,000 bootstrap replicates under Kimura’s two-parameter correction using Geneious 7.1.6 [34] and the results are presented in Figs 1 and 2.

### Comparison of fungal growth in culture

The 67 fungal isolates collected from strawberry in the UK were compared with a subset of other isolates (chosen based on genetic, host and geographic diversity) including 49 isolates of *C*. *acutatum s*.*l*. and four isolates of *C*. *gloeosporioides s*.*l*. for *in vitro* growth studies on PDA (Potato Dextrose Agar, BD Difco). For experiments, a 7 mm diameter mycelial plug excised from the edge of an actively growing PDA culture was placed at the centre of a fresh PDA plate. In the growth experiment, two perpendicular colony diameters were measured daily and colony radius was calculated from cultures incubated at four different temperatures (15°C, 20°C, 25°C and 30°C) in darkness. Data corresponding to the linear growth phase were subjected to analysis of variance of regression in order to create growth curves for each isolate at each temperature. In both tests three plates were used as replicates. Statistical analysis was performed by SIGMAPLOT 10 program (Sigmaplot Software, USA). Colony characters were recorded after 15 days of incubation at 25°C under 12 h light/ 12 h dark cycle.

### Pathogenicity tests

**Fig 3:**
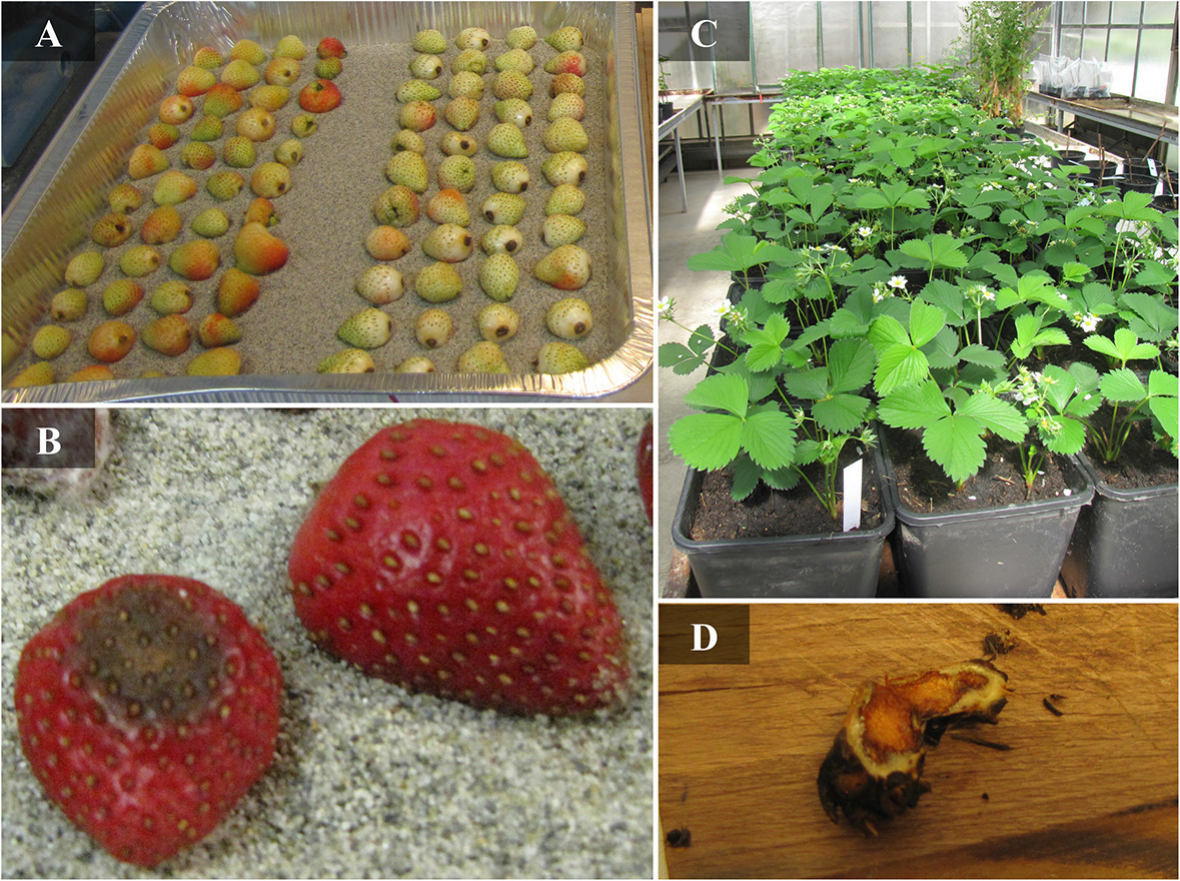
Strawberry fruits and plants used for pathogenicity tests (A and C) and symptoms (B and D). **(**A) Unripe fruits (phenological stage turning white-pink) used for artificial inoculations of *Colletotrichum* spp. (B) Strawberry fruits 7 days after inoculation with *Colletotrichum* sp. spores suspension showing typical black spot symptoms (bottom left) and with sterile water used as control (top right) (C) Three-month-old strawberry plants used to pathogenicity assays (D) Strawberry plant crown sectioned showing presence of red-brownish lesions characteristic of anthracnose caused by *Colletotrichum* spp.

Representative isolates (highlighted with asterisks in Table 1) of each *C*. *acutatum s*.*l*. group isolated from strawberry in UK, together with reference isolates from other hosts, were used for pathogenicity tests on the generally susceptible strawberry cultivar Elsanta [38]. A conidial suspension was prepared for each isolates by flooding 10-day-old PDA culture plates with sterile deionised water. Spore concentration was adjusted to 10^5^ spores ml^-1^ and 10^6^ spores ml^-1^ for fruit and crown inoculation, respectively [7,38]. Unripe fruits (white fruit beginning to turn pink, as shown in Fig 3A) [39] were inoculated with a 5μl drop of conidial suspension. Before inoculation, fruit surfaces were disinfected for 5 min using NaClO (1% active chlorine) in 50% EtOH, washed three times in sterilized water, blotted dry and placed in a tray with moist sand on the bottom to prevent movement of the fruits during further procedures. After inoculation, fruits were incubated at 25°C under 12h light/ 12h in dark cycle.

Disease symptoms were evaluated 7 days after inoculation (d.a.i.) (Fig 3B) by recording the incidence of disease (% of infected fruits), and the aggressiveness of lesion development using the following severity scale: 0, no visible lesions; 1, lesions on less than 33% of fruit surface; 2, lesions covering 33–66% of fruit surface; and 3, lesions covering more than 66% of fruit surface. Three fruits inoculated with sterile distilled water (SDW) as well as fresh fruits served as non-inoculated controls. Four independent replicates were tested for each fungal isolate, consisting of three inoculated fruits for each replicate. At the end of the experiment, *Colletotrichum* isolates were re-isolated from infected fruits and cultured on PDA to confirm colony characteristics.

The capability of the isolates to produce crown rot symptoms was evaluated by injecting the crowns of three-months-old strawberry plants (Fig 3C) with 0.2 mL conidial suspension using a syringe [4,7]. Plants were placed in glasshouse at 23°C with 16h light / 8h darkness. After 24 days (d.a.i.), plants were evaluated for the presence of crown tissues with red-brownish discoloration, wilting and collapse of the plant, typical symptoms of *Colletotrichum* crown rot, according to the following severity scale: 0, no lesions; 1, crown tissues discoloration but no wilting or collapse; 2, wilting or collapse of part of the plant; and 3, plant death. Crowns of all plants were sectioned and examined for the presence of red-brownish lesions (Fig 3D). Crown infection was confirmed by re-isolation of the pathogen. Three plant crowns injected with SDW as well as untouched plants served as negative controls for each replicates. The experiment was independently replicated three times, with six plants for each replicate.

Values of disease severity were used to calculate a Disease Index (DI, average severity) according to the following formula: Σvn/N, where v represents the numeric value of the class, n is the number of plants or fruits assigned to the class, N is the total number of the plants or fruits assessed. Data for pathogenicity tests on both fruits and plants were subjected to analysis of variance ANOVA and means compared using Tukey’s multiple range test by Systat11 (Systat Software, USA).

## Results

### Characterization of genetic variation, and species identification

Phylogenetic trees were constructed using combined ITS, TUB2, GADPH and MAT1-2 sequence data set consisting of 148 *Colletotrichum* isolates (Table 1). As shown in Fig 1, most of the *C*. *acutatum s*.*l*. isolates (49/67) were identified as belonging to *C*. *nymphaeae* (= A2 genetic group), based on clustering with high bootstrap value with the reference isolates CBS 797.72, PT135, IMI345028 and other genetically similar isolates (identical sites = 1422/1438 or 98.9%; pairwise identity = 99.9%). A smaller proportion of isolates in the diversity collection (12/67) were identified as belonging to *C*. *godetiae* (= A4 genetic group) based on genetic clustering with reference isolates ALMNRB-30K, CBS 193.32 and JL198 (identical sites = 1411/1438 or 94.6%; pairwise identity = 99.4%). And finally, six isolates were identified as belonging to *C*. *fioriniae* (= A3 genetic group) based on clustering with the reference isolate ATCC 56813 (identical sites = 1.436 /1443 or 99.5%; pairwise identity = 99.9%).

Molecular characterisation of 67 *Colletotrichum* isolates collected from strawberry in the UK along with the reference isolates representing the host and geographic diversity (Figs 1 and 2) suggests that there have been multiple introductions of the anthracnose pathogen belonging to different *Colletotrichu*m species into the country. Three different species *C*. *nymphaeae*, *C*. *godetiae* and *C*. *fioriniae* were identified based on sequence from four loci [6,17,18]. Incidence of these species is shown in Fig 2, where *C*. *nymphaeae* corresponds to 73%, followed by *C*. *godetiae* (18%) and *C*. *fioriniae* (9%). GAPDH is the locus that shows the highest variability across the nucleotide dataset, with 24.1% identical sites for the entire set of data (out-group included) and 59.3% within *C*. *acutatum s*.*l*. The MAT1-2 gene also shows a high variability with 34.4% identical sites of which 78.6% in *C*. *acutatum s*.*l*. TUB and ITS loci show lower percentage of variable sites. In detail, TUB has 58.1% of identical sites in the final alignment and 80.7% only considering *C*. *acutatum s*.*l*. While ITS has 77.8% and 92.4% of conserved nucleotides, respectively with and without out-groups. Based on the nucleotide variability referred to above, four haplotypes of *C*. *nymphaeae*, three haplotypes of *C*. *fioriniae*, and five haplotypes of *C*. *godetiae* were identified further highlighting the multiple introductions of the pathogens belonging to these species into the UK.

### Fungal growth in plate culture

Radial growth data of *C*. *acutatum s*.*l*. and *C*. *gloeosporioides s*.*l*. isolates were subjected to analysis of variance of regression in order to obtain growth curves that were all statistically significant (R^2^≥0.9447 and P<0.0001), with the only exception of one isolate showing a R^2^ = 0.770 (*C*. *nymphaeae* CSL224 at 30°C). The slope for each isolate (three replicates for each isolate) belonging to the same species were averaged, in order to detect the hypothetical optimal growth temperature, and results are shown in Table 2. Almost all species, particularly those containing isolates from strawberry in the UK namely *C*. *nymphaeae*, *C*. *fioriniae*, and *C*. *godetiae* had highest growth rates at 25°C that was considered as optimum temperature. It is pertinent to mention that higher levels of strawberry anthracnose incidence in the UK have been reported in the southwest and southeast regions, where relatively high temperatures are most often reached [20]. However, *C*. *phormii*, *C*. *kinghormii* and *C*. *rhombiforme* showed the highest growth rate at the temperature of 20°C and they were not able to grow at 30°C. Interestingly, these three species are evolutionarily closely related, suggesting a specific adaptation to different environmental conditions compared to other members of the same complex. With respect to *C*. *gloeosporioides s*.*l*. isolates (*C*. *aenigma* CSL780 and CSL 869; *C*. *fruticola* CSL 311 and CSL386), used as out-groups, all the four isolates showed the highest growth rate at all the tested temperatures when compared with all the other isolates.

**Table 2:** Radial growth rate (mm h^-1^) of each *Colletotrichum* species at different temperatures.

	Species	15°C*	20°C*	25°C*	30°C*
out-group	***C*. *aenigma***	0.112 ± 0.001	0.199 ± 0.002	**0.261 ± 0.008**	0.124 ± 0.011
	***C*. *fruticola***	0.118 ± 0.004	0.209 ± 0.005	**0.238 ± 0.019**	0.150 ± 0.008
*Colletotrichum acutatum* species complex	***C*. *rhombiforme***	0.091 ± 0.001	**0.135 ± 0.001**	0.111 ± 0.002	0.000 ± 0.000
	***C*. *kinghornii***	0.073 ± 0.001	**0.108 ± 0.001**	0.077 ± 0.002	0.000 ± 0.000
	***C*. *phormii***	0.106 ± 0.001	**0.166 ± 0.001**	0.139 ± 0.002	0.000 ± 0.000
	***C*. *salicis***	0.094 ± 0.001	0.147 ± 0.002	**0.179 ± 0.004**	0.035 ± 0.005
	***C*. *godetiae***	0.094 ± 0.002	0.142 ± 0.003	**0.163 ± 0.005**	0.004 ± 0.000
	***C*. *acutatum***	0.054 ± 0.004	0.087 ± 0.006	**0.148 ± 0.004**	0.058 ± 0.005
	***C*. *fioriniae***	0.081 ± 0.003	0.136 ± 0.005	**0.185 ± 0.004**	0.083 ± 0.006
	***Colletotrichum* sp. 2**	0.085 ± 0.002	0.140 ± 0.001	**0.178 ± 0.002**	0.075 ± 0.002
	***C*. *lupini***	0.086 ± 0.001	0.130 ± 0.003	**0.152 ± 0.009**	0.058 ± 0.002
	***Colletotrichum* sp. 1**	0.083 ± 0.001	0.132 ± 0.001	**0.138 ± 0.001**	0.043 ± 0.001
	***C*. *tamarilloi***	0.069 ± 0.001	0.123 ± 0.002	**0.148 ± 0.003**	0.007 ± 0.000
	***C*. *simmondsii***	0.040 ± 0.003	0.092 ± 0.008	**0.112 ± 0.014**	0.089 ± 0.010
	***C*. *laticiphilum***	0.058 ± 0.002	0.113 ± 0.001	**0.161 ± 0.001**	0.121 ± 0.002
	***C*. *nymphaeae***	0.077 ± 0.001	0.135 ± 0.002	**0.159 ± 0.004**	0.063 ± 0.005
	***C*. *chrysanthemi***	0.050 ± 0.001	0.083 ± 0.001	**0.111 ± 0.001**	0.087 ± 0.002
	***C*. *scovillei***	0.036 ± 0.001	0.105 ± 0.001	**0.115 ± 0.001**	0.062 ± 0.002

* Values represent the average + SD of slopes (growth rates expressed as mm h-1) of all isolates belonging to the same species, three replicates for each isolate. The optimal temperature for each species is indicated in bold.

*C*. *nymphaeae* isolates developed white cottony aerial mycelium, light brownish conidial masses with peculiar colony colour from dark grey to dark brown. Twelve isolates belonging to *C*. *godetiae* were characterized by white aerial mycelium, and yellow pigmentation to white colour on the reverse side of the culture. *C*. *fioriniae* isolates were dark red on the reverse side of the cultures with orange conidial masses in large drops on the colony surface, and conidiomata formed directly on the hyphae. However, these characters are often difficult to describe reliably, and can change following sub-culturing or based on the length and type of storage. Thus, there is a need for further development of molecular methods for reliable and rapid diagnosis and monitoring of the pathogen populations belonging to different species associated with strawberry production in a specific geographic location.

### Characterisation of variation in pathogenicity

Thirty-four *C*. *acutatum s*.*l*. isolates were chosen for pathogenicity tests on fruits and plants, including six representative isolates from each of the three species described above related to strawberry production in the UK (highlighted with * in Table 1 and in bold in Fig 1), and one or more isolates representative of all the major species of the *C*. *acutatum* complex. Four *C*. *gloeosporioides s*.*l*. isolates that were isolated from strawberry infected tissues from UK (CSL 780 and CSL 869, *C*. *aenigma*) and USA (CSL 311 and CSL 386, *C*. *fruticola*) were included in the experiments as an out-group.

*C*. *acutatum s*.*l*. isolates varied in aggressiveness on both host tissues. In the fruit assays, among the three species identified from the strawberry production systems in the UK, *C*. *nymphaeae* and *C*. *fioriniae* were more aggressive compared to *C*. *godetiae*. This was particularly noticeable for isolates originating from strawberry as reflected by the fruit disease index range for *C*. *nymphaeae* (2.08–3.00), *C*. *fioriniae* (1.92–2.75) and *C*. *godetiae* (0.75–2.08). Interestingly, with isolates originating from other hosts, *C*. *nymphaeae* isolates were less aggressive (0.67–1.67), and one or more isolates belonging to *C*. *fioriniae* (2.00–2.17) as well as *C*. *godetiae* (2.17) showed fruit disease index in the range of the strawberry isolates. Among the other species tested within the *C*. *acutatum* complex, *C*. *acutatum* s.s., *C*. *simmondsii* and *Colletotrichum* sp.2 included one or more isolates originating from non-strawberry hosts that showed medium level of aggressiveness with fruit disease index ranging from 1.17 to 2.08. Whereas, *C*. *lupini* (0.08–075), *C*. *phormii* (0.58), *C*. *salicis* (0.17–0.67), and *C*. *rhombiforme* (0.67) along with *Colletotrichum* sp.1 (0.33) isolates originating from various hosts other than strawberry were much less aggressive as reflected by the fruit disease index. The *C*. *gloeosporioides s*.*l*. isolates tested showed a fruit disease index ranging from 1.50 to 2.50 (Table 3).

**Table 3:** Variability in aggressiveness of *Colletotrichum* species isolates on strawberry fruits and plants.

	Isolate	Species	Isolation source	Origin	Fruit Disease index^*^ ^,^ ^+^		Plant Disease Index^*^ ^,^ ^#^	
*Colletotrichum acutatum* species complex	CSL 256	*C*. *nymphaeae*	*Fragraria*	UK	2.50	abcd	0.50	bc
	CSL 899	*C*. *nymphaeae*	*Fragraria*	UK	3.00	a	0.83	abc
	CSL 915	*C*. *nymphaeae*	*Fragraria*	UK	2.08	abcdef	0.61	bc
	ATCC 38896	*C*. *nymphaeae*	*Nymphaeae*	Netherlands	0.67	defg	0.28	bc
	CSL 455	*C*. *nymphaeae*	*Photinia*	UK	1.08	bcdefg	0.56	bc
	PT135	*C*. *nymphaeae*	*Olea*	Portugal	1.67	abcdefg	0.89	abc
	CSL 916	*C*. *godetiae*	*Fragraria*	UK	1.92	abcdefg	0.39	bc
	CSL 918	*C*. *godetiae*	*Fragraria*	UK	0.75	cdefg	0.39	bc
	CSL 919	*C*. *godetiae*	*Fragraria*	UK	2.08	abcdef	0.67	bc
	ALM-NRB-30K	*C*. *godetiae*	*Prunus*	Israel	0.25	fg	0.11	c
	CBS 193.32	*C*. *godetiae*	*Olea*	Greece	0.75	cdefg	0.28	bc
	JL198	*C*. *godetiae*	*Olea*	Serbia	2.17	abcde	0.39	bc
	CSL 1259	*C*. *fiorinae*	*Fragraria*	UK	2.75	ab	0.72	bc
	CSL 1262	*C*. *fiorinae*	*Fragraria*	UK	1.92	abcdefg	1.00	ab
	CSL 2546	*C*. *fiorinae*	*Fragraria*	UK	2.67	abc	0.72	bc
	CBS 797.72	*C*. *fiorinae*	*Pinus*	New Zealand	1.08	bcdefg	0.39	bc
	ATCC MYA-663	*C*. *fiorinae*	*Malus*	USA	2.00	abcdef	0.83	abc
	CR46	*C*. *fiorinae*	*Vitis*	Portugal	2.17	abcde	0.33	bc
	PJ8	*C*. *acutatum*	*Pyrus*	New Zealand	2.08	abcdef	0.72	bc
	PT227	*C*. *acutatum*	*Olea*	Portugal	1.42	abcdefg	0.78	abc
	STE-U-164	*C*. *acutatum*	*Pinus*	South Africa	0.83	cdefg	0.28	bc
	CBS 122122	*C*. *simmondsii*	*Carica*	Australia	0.25	efg	0.22	bc
	CBS 294.67	*C*. *simmondsii*	*Carica*	Australia	1.17	abcdefg	0.61	bc
	PD89-582	*C*. *simmondsii*	*Cyclamen*	Netherland	1.83	abcdefg	0.44	bc
	BBA 70884	*C*. *lupini*	*Lupinus*	Ukraine	0.58	efg	0.33	bc
	HY09	*C*. *lupini*	*Lupinus*	Canada	0.08	g	0.17	bc
	PT30	*C*. *lupini*	*Lupinus*	Portugal	0.75	cdefg	0.56	bc
	9178	*C*. *salicis*	*Vaccinium*	Norway	0.50	efg	0.28	bc
	CBS 607.94	*C*. *salicis*	*Salix*	Netherlands	0.67	defg	0.17	bc
	MP1	*C*. *salicis*	*Acer*	USA	0.17	fg	0.22	bc
	CBS 101611	*Colletotrichum* sp. 1	*Fern*	Costa Rica	0.33	efg	0.06	c
	OCO-ARC-4	*Colletotrichum* sp. 2	*Citrus*	USA	1.42	abcdefg	0.11	c
	AR3787	*C*. *phormii*	*Phormium*	South Africa	0.58	efg	0.22	bc
	PT250	*C*. *rhombiforme*	*Olea*	Portugal	0.67	defg	0.33	bc
out-group	CSL 780	*C*. *aenigma*	*Fragraria*	UK	2.50	abcd	0.50	bc
	CSL 869	*C*. *aenigma*	*Fragraria*	UK	1.92	abcdefg	0.72	bc
	CSL 311	*C*. *fruticola*	*Fragraria*	USA	2.50	abcd	1.56	a
	CSL 386	*C*. *fruticola*	*Fragraria*	USA	1.50	abcdefg	0.22	bc

Disease Index data related to aggressiveness on strawberry fruits and crowns of representative *Colletotrichum* isolates.

*: Different letters within the same column correspond to significantly different values (ANOVA; P < 0.05). The values are the averages ± SD of four independent replicates, three fruits for each replicate and of three independent replicates, six plants for each replicate. Disease Index was calculated according to the following formula: Σvn/N, where v represents the numeric value of the class, n is the number of fruits or plants assigned to the class, N is the total number of the plants assessed.

+: 0, no visible lesions; 1, lesions on less than 33% of fruit surface; 2, lesions covering 33–66% of fruit surface; and 3, lesions covering more than 66% of fruit surface.

#: 0, no lesions; 1, crown tissues discoloration but no wilting or collapse; 2, wilting or collapse of part of the plant; and 3, plant death.

In the *in vitro* assays, anthracnose fruit rot symptoms were observed (e.g. Fig 3B) for various isolates tested with different levels of aggressiveness, as shown by the disease index ranging from 0.08 to 3.0 (Table 3). The variation in aggressiveness among different isolates was clearly reflected by the differences in incidence which ranged from 8.33 to 100% with only 4 out of 38 isolates showing 91.7 to 100% as well as the lesion type which ranged from 0.1 to 3.0 (S1 Table). When lesion morphology was evaluated, different kinds of lesions could be distinguished on fruits, ranging from brown ones containing orange drops of conidia to those entirely covered with aerial mycelium, with different lesion size. *C*. *nymphaeae* CSL899 was the most aggressive on strawberry fruits with the highest disease index (3.0, corresponding to symptoms covering more than 66% of fruit surface).

In the plant assays, varying degrees of crown rot symptoms were recorded 24 d.a.i, as reflected by the disease index range shown in Table 3. Symptom severity was generally low, with no isolate scoring higher than 2 (wilting and collapse of plant). Among the three species identified from UK strawberry production systems, *C*. *fioriniae* isolates originating from strawberry showed a higher range of disease index (0.72–1.00) compared to *C*. *nymphaeae* (0.5–0.83) and *C*. *godetiae* (0.39–0.67). The *C*. *gloeosporioides s*.*l*. isolate CSL 311 (*C*. *fruticola* from strawberry in USA) showed the highest disease index (1.6), this isolate was also amongst the most aggressive on fruit (Table 3). *Colletotrichum* isolates were recovered from all crowns showing symptoms.

## Discussion

The UK strawberry industry has expanded rapidly in recent years, and this appears to correlate with increasing losses attributed to anthracnose caused by *Colletotrichum spp*. [6]. This study provides the first molecular characterization of *C*. *acutatum sensu lato* diversity related to strawberry production in the UK, combined with pathogenic characterization. A collection of 148 isolates representative of UK and global diversity of *C*. *acutatum s*.*l*. populations has been assembled. The isolates were chosen based on host association, geographic distribution, phylogenetic relationships and biological diversity.

On the basis of four sequence loci (ITS, TUB, GAPDH, and MAT1-2), the *C*. *acutatum sensu lato* isolates were assigned to three newly designated species *C*. *nymphaeae*, *C*. *godetiae* and *C*. *fioriniae* following a recent taxonomic re-assessment [18]. According to available literature, *C*. *nymphaeae* is the most common and *C*. *godetiae* is also often reported in European and American strawberry fields [6]. These two species were also the most representative in our dataset of isolates related to strawberry in the UK. *C*. *fioriniae* has a worldwide distribution and is common on strawberry but only a few isolates were identified in our collection, and this group was not commonly present in the fields in the UK. *C*. *simmondsii*, *C*. *acutatum sensu stricto*, *C*. *salicis* and *C*. *miyabeana* are common on strawberry in Oceania and have only been found sporadically in Europe. Isolates belonging to these species have not been detected on strawberry in the UK. The variability observed within the UK *C*. *acutatum sensu lato* species fits in part with previous reports of *C*. *acutatum* on strawberry within specific geographic regions. For example, in France, Israel, Bulgaria and Spain, the majority of strawberry anthracnose pathogen isolates clustered in the same species *C*. *nymphaeae*, and almost no intra-specific diversity was observed within each country [2–5]. A different situation has been observed on Belgian isolates, where the population represented: 33% isolates belonging to *C*. *nymphaeae*, 5% *C*. *fioriniae*, 50% *C*. *godetiae*, 3% *C*. *acutatum s*.*s*. and 6% *C*. *salicis*. A possible explanation to *C*. *acutatum s*.*l*. status in the UK might be recent introduction (late 70s) from a limited number of sources. The reason for the differences in the occurrence of various *Colletotrichum* species associated with strawberry production in different geographic locations still remains unclear, but the source of importation of the planting material and local trade have been heavily implicated [4,7].

The pathogenicity assays used in this work are based on a study in Belgium [7] in view of the similar molecular diversity of the anthracnose pathogen populations associated with strawberry production. These assays with the isolates representing the molecular diversity not only revealed variability in aggressiveness in different species described within *C*. *acutatum s*.*l*., but also complex patterns both between and within the species. For example, based on isolates originating from strawberry, *C*. *fioriniae* and *C*. *nymphaeae* appear equally aggressive on fruits with *C*. *nymphaeae* isolates indicating a degree of host-preference. Both *C*. *fioriniae* and *C*. *godetiae* included isolates originating from other hosts that showed comparable levels of aggressiveness to isolates from strawberry. Similar situation was observed with at least some non-strawberry isolates belonging to species such as *C*. *acutatum s*.*s*. and *C*. *simmondsii*. Furthermore, at least one *C*. *godetiae* isolate from strawberry was much less aggressive compared to others. These patterns suggest that some *Colletotrichum* species such as *C*. *fioriniae* and *C*. *godetiae* include populations that are capable of infecting a wider range of hosts, also influenced by environmental conditions. Further studies using a wider set of isolates of these three species and appropriate pathological and biological assays are required to gain additional insights into the evolution of pathogenicity in relation to field symptoms as well as any differential responses to host varieties and fungicides locally used in the UK strawberry production systems.

The study has highlighted the genetic and pathogenic heterogeneity of the introduced anthracnose pathogen populations belonging to three different *Colletotrichum* species emphasising the need for effective phytosanitary procedures linked to pathogen monitoring and characterisation to generally limit the entry of non-native pathogens. This also underlines the requirement of reliable and rapid diagnostic tools for further research and application in strawberry anthracnose management. The recent release of a whole genome sequence of *C*. *fioriniae* isolated from strawberry [28] along with the newly characterised isolates, based on multi-locus sequence and aggressiveness information reported here, represents a useful platform for further research into the genetic basis of *C*. *acutatum s*.*l*.—strawberry interactions.

## Supporting Information

S1 Table Variability in aggressiveness of Colletotrichum species isolates on strawberry fruits and plants. ^a^ 0, no visible lesions; 1, lesions on less than 33% of fruit surface; 2, lesions covering 33–66% of fruit surface; and 3, lesions covering more than 66% of fruit surface. ^b^ no lesions; 1, crown tissues discoloration but no wilting or collapse; 2, wilting or collapse of part of the plant; and 3, plant death.
(XLSX)
